# Supplementary material for: PCSK9 genetic variants and risk of vascular and non-vascular diseases in Chinese and UK populations
Source: Eur J Prev Cardiol. 2024 Jan 10;31(8):1015–25. doi: 10.1093/eurjpc/zwae009 (PMC11144468; doi:10.1093/eurjpc/zwae009)
Supplement: Supplementary file 1 [file zwae009_supplementary_data.pdf]

Supplemental Material

PCSK9 genetic variants and risk of vascular and non-vascular diseases in Chinese and UK populations

Michael V Holmes*, Christiana Kartsonaki*, Ruth Boxall*, Kuang Lin, Nicola Reeve, Canqing Yu, Jun Lv, Derrick A Bennett, Michael R Hill, Ling Yang, Yiping Chen, Huaidong Du, Iain Turnbull, Rory Collins, Robert J Clarke, Martin Tobin, Liming Li, Iona Y Millwood#, Zhengming Chen#, Robin G Walters#, on behalf of the China Kadoorie Biobank Collaborative Group

Table of Contents

Members of the China Kadoorie Biobank collaborative group: 4

Supplementary Methods 5

Derivation of the LDL cholesterol genetic risk score 5

Replication of COPD exacerbation in UK Biobank 5

Supplementary Tables 6

Supplementary Table 1: Association of SNPs included in the PCSK9 genetic score with LDL-cholesterol in univariate and multivariate analysis. 6

Supplementary Table 2: Association of SNPs included in the *PCSK9* genetic score with LDL-cholesterol in East Asian populations according to Global Lipids Genetics Consortium. 7

Supplementary Table 3: Associations with LDL-cholesterol in GLGC and CKB of 74 previously identified LDL-cholesterol-associated SNPs 8

Supplementary Table 4: ICD-10 codes of vascular and non-vascular endpoints in CKB 11

Supplementary Table 5: ICD-10 codes of endpoints included in the phenome scan 12

Supplementary Table 6: Numbers and ascertainments of cases and controls included in analyses of disease associations with the *PCSK9* genetic score 14

Supplementary Table 7: Disease definitions for replication of outcomes reported as associated with a functional *PCSK9* variant in UK Biobank 17

Supplementary Table 8: Association of the *PCSK9* genetic score with carotid intima media thickness and presence of carotid plaque in CKB second resurvey participants 18

Supplementary Table 9: Association of the *PCSK9* genetic score with FEV1/FVC measured at baseline in CKB participants 19

Supplementary Table 10: Association of a loss-of-function *PCSK9* SNP with exacerbations of COPD in UK Biobank 20

Supplementary Figures 21

Supplementary Figure 1. Study design flow-chart. 21

Supplementary Figure 2. Violin plots showing the distribution of measured FEV1/FVC in each CKB region 22

Supplementary Figure 3. Histogram of measured FEV1/FVC by prevalent COPD status. 23

Supplementary Figure 4: Frequency distribution of the *PCSK9* genetic score in the CKB population-representative subset. 24

Supplementary Figure 6: Association of *PCSK9* genetic score with metabolic biomarkers quantified by ^{1}H-NMR spectroscopy...26
Supplementary Figure 7: Association of *PCSK9* genetic score with non-lipid blood measures 27
Supplementary Figure 8: Phenome-wide scan of *PCSK9* genetic score associations in China Kadoorie Biobank 28
Supplementary Figure 9: Associations of *PCSK9*-GS with respiratory disease endpoints in CKB, with prevalent COPD defined as FEV1/FVC<0.7. 29
Supplementary Figure 10: Sub-group analyses of association of *PCSK9* genetic score with upper respiratory tract infections...30

Members of the China Kadoorie Biobank collaborative group:

International Steering Committee: Junshi Chen, Zhengming Chen (PI), Robert Clarke, Rory Collins, Yu Guo, Liming Li (PI), Chen Wang, Jun Lv, Richard Peto, Robin Walters.

International Co-ordinating Centre, Oxford: Daniel Avery, Derrick Bennett, Ruth Boxall, Sushila Burgess, Ka Hung Chan, Yiping Chen, Zhengming Chen, Johnathan Clarke; Robert Clarke, Huaidong Du, Ahmed Edris, Hannah Fry, Simon Gilbert, Mike Hill, Pek Kei Im, Andri Iona, Maria Kakkoura, Christiana Kartsonaki, Hubert Lam, Kuang Lin, Mohsen Mazidi, Iona Millwood, Sam Morris, Qunhua Nie, Alfred Pozarickij, Paul Ryder, Saredo Said, Dan Schmidt, Paul Sherliker, Becky Stevens, Iain Turnbull, Robin Walters, Baihan Wang, Lin Wang, Neil Wright, Ling Yang, Xiaoming Yang, Pang Yao.

National Co-ordinating Centre, Beijing: Xiao Han, Can Hou, Qingmei Xia, Chao Liu, Jun Lv, Pei Pei, Canqing Yu.

Regional Co-ordinating Centres:

Gansu: Gansu Provincial CDC – Caixia Dong, Pengfei Ge, Xiaolan Ren. Maiji CDC – Zhongxiao Li, Enke Mao, Tao Wang, Hui Zhang, Xi Zhang. **Haikou:** Hainan Provincial CDC – Jinyan Chen, Ximin Hu, Xiaohuan Wang. Meilan CDC – Zhendong Guo, Huimei Li, Yilei Li, Min Weng, Shukuan Wu. **Harbin:** Heilongjiang Provincial CDC – Shichun Yan, Mingyuan Zou, Xue Zhou. Nangang CDC – Ziyan Guo, Quan Kang, Yanjie Li, Bo Yu, Qinai Xu. **Henan:** Henan Provincial CDC – Liang Chang, Lei Fan, Shixian Feng, Ding Zhang, Gang Zhou. Huixian CDC – Yulian Gao, Tianyou He, Pan He, Chen Hu, Huarong Sun, Xukui Zhang. **Hunan:** Hunan Provincial CDC – Biyun Chen, Zhongxi Fu, Yuelong Huang, Huilin Liu, Qiaohua Xu, Li Yin. Liuyang CDC – Huajun Long, Xin Xu, Hao Zhang, Libo Zhang. **Liuzhou:** Guangxi Provincial CDC – Naying Chen, Duo Liu, Zhenzhu Tang. Liuzhou CDC – Ningyu Chen, Qilian Jiang, Jian Lan, Mingqiang Li, Yun Liu, Fanwen Meng, Jinhuai Meng, Rong Pan, Yulu Qin, Ping Wang, Sisi Wang, Liuping Wei, Liyuan Zhou. **Qingdao:** Qingdao CDC – Liang Cheng, Ranran Du, Ruqin Gao, Feifei Li, Shanpeng Li, Yongmei Liu, Feng Ning, Zengchang Pang, Xiaohui Sun, Xiaocao Tian, Shaojie Wang, Yaoming Zhai, Hua Zhang, Licang CDC – Wei Hou, Silu Lv, Junzheng Wang. **Sichuan:** Sichuan Provincial CDC – Xiaofang Chen, Xianping Wu, Ningmei Zhang, Weiwei Zhou. Pengzhou CDC – Xiaofang Chen, Jianguo Li, Jiaqiu Liu, Guojin Luo, Qiang Sun, Xunfu Zhong. **Suzhou:** Jiangsu Provincial CDC – Jian Su, Ran Tao, Ming Wu, Jie Yang, Jinyi Zhou, Yonglin Zhou. Suzhou CDC – Yihe Hu, Yujie Hua, Jianrong Jin Fang Liu, Jingchao Liu, Yan Lu, Liangcai Ma, Aiyu Tang, Jun Zhang. **Zhejiang**: Zhejiang Provincial CDC – Weiwei Gong, Ruying Hu, Hao Wang, Meng Wang, Min Yu. Tongxiang CDC – Lingli Chen, Qijun Gu, Dongxia Pan, Chunmei Wang, Kaixu Xie, Xiaoyi Zhang.

Supplementary Methods

Derivation of the LDL cholesterol genetic risk score

The LDL cholesterol (LDL-C) genetic risk score (GS) was constructed by using single nucleotide polymorphisms (SNPs) previously identified as associated with LDL-C at $P<5x10^{-8}$ by the Global Lipid Genetics Consortium (GLGC)[36]. Prior to inclusion of each SNP in the LDL-C GS, we assessed heterogeneity of the per-allele LDL-C effect estimate between CKB and GLGC using Cochran's Q statistic; SNPs without evidence of heterogeneity between CKB and GLGC (after Bonferroni multiple testing correction for 74 SNPs) were weighted using GLGC association estimates. SNPs with effect sizes in CKB significantly different from the estimate from GLGC were excluded from the instrument if (i) they did not show an association with LDL-C in CKB (at P<0.05/74) or (ii) if the direction of association was opposite to that reported in GLGC. The association of the weighted LDL-C GS with disease outcomes was assessed by logistic regression, stratified by region, and with adjustment for age, age^2, sex and 2-9 regional genetic principal components.

Replication of COPD exacerbation in UK Biobank

To replicate the association of *PCSK9* variants with COPD exacerbations, we obtained estimates of the per-allele estimate of a functional *PCSK9* variant (rs11591147) with risk of COPD exacerbations defined as the presence of one or more acute exacerbations from HES data among 25,030 participants in the UK Biobank with COPD GOLD 2-4 (defined as FEV1/FVC<0.7 and percent predicted FEV1<80%) at study baseline. The estimate was scaled to an equivalent per-1-SD lower LDL-C using the effect estimate from GLGC, and was compared with our estimate from CKB. UK Biobank analyses were performed under project approval 648.

Supplementary Tables

Supplementary Table 1: Association of SNPs included in the PCSK9 genetic score with LDL-cholesterol in univariate and multivariate analysis.

			Univariate Model				Multivariate Model			
**SNP**	**Effect/ other allele**	**Effect allele freq.**	**N**	**effect (SD)**	**SE**	**P-value**	**N**	**effect (SD)**	**SE**	**P-value**
rs151193009	C/T	0.986	16913	0.630	0.047	3.0E-41	16913	0.646	0.047	3.2E-43
rs2495477	A/G	0.738	16913	0.058	0.013	1.0E-05	16913	0.104	0.014	1.2E-13
rs11206517	G/T	0.060	16913	0.102	0.023	9.9E-06	16913	0.159	0.025	8.7E-11

Analyses were stratified by region and combined by inverse-variance-weighted fixed effect meta-analysis. LDL-cholesterol measures from clinical biochemistry assays from each region were adjusted for age, age^2, fasting time, fasting $time^2$, ascertainment group, sex, and 2-9 regional principal components, and rank inverse normal transformed. Effect allele is defined as the LDL-C increasing allele.

Supplementary Table 2: Association of SNPs included in the *PCSK9* genetic score with LDL-cholesterol in East Asian populations according to Global Lipids Genetics Consortium.

			Univariate Model[1]			
**SNP**	**Effect/ other allele**	**Effect allele freq.**	**N**	**effect (SD)**	**SE**	**P-value**
rs151193009	C/T	0.991	50235	0.486	0.033	2.34E-44
rs2495477	A/G	0.756	79693	0.050	0.006	6.77E-14
rs11206517	G/T	0.050	82587	0.081	0.012	2.69E-11

[1]Summary statistics from Graham, *et al.* (2023). Nature **600**, 675-679

Supplementary Table 3: Associations with LDL-cholesterol in GLGC and CKB of 74 previously identified LDL-cholesterol-associated SNPs

						CKB Univariate Model				GLGC (As Used for Score)					
rsID	Chr:Pos	Genes	Effect/ other allele	EAF (CKB)	EAF (1KG)	N	Estimate (SD)	SE	P-value	N	Estimate (SD)	SE	P-value	Het P-value	Included in CKB LDL-C GS?
rs10903129	1:25641524	TMEM57	G/A	0.300	0.227	16913	0.035	0.012	0.003	169920	0.033	0.004	4.0E-19	0.885	Yes
rs4587594	1:62906518	DOCK7	G/A	0.812	0.823	16913	0.019	0.014	0.175	173007	0.049	0.004	3.0E-37	0.034	Yes
rs6603981	1:92766395	EVI5	T/C	0.960	0.975	16913	0.067	0.029	0.019	173056	0.034	0.004	2.0E-14	0.252	Yes
rs646776	1:109620053	*CELSR2/MYBPHL/PSRC1/SARS/SORT1	T/C	0.941	0.954	16913	0.213	0.023	9.6E-21	173021	0.160	0.004	1.0E-292	0.022	Yes
rs267733	1:149225460	ANXA9	A/G	0.964	0.974	16913	-0.030	0.028	0.296	164562	0.033	0.005	5.0E-10	0.030	Yes
rs2642438	1:219036651	MOSC1	G/A	0.829	0.768	16913	0.065	0.015	1.2E-05	165470	0.035	0.004	4.0E-17	0.053	Yes
rs903319	1:219052434	MOSC1	C/T	0.297	0.299	16913	0.012	0.012	0.326	172998	0.027	0.004	8.0E-11	0.244	Yes
rs2587534	1:232915962	CR596412	A/G	0.761	0.769	16913	0.060	0.013	5.0E-06	172966	0.039	0.004	3.0E-26	0.130	Yes
rs1367117	2:21117405	APOB	A/G	0.126	0.115	16913	0.099	0.016	1.9E-09	173007	0.119	0.004	2.0E-196	0.236	Yes
rs3817588	2:27584716	GCKR	T/C	0.668	0.657	16913	0.034	0.012	0.004	172990	0.026	0.005	3.0E-08	0.511	Yes
rs6544713	2:43927385	ABCG8	T/C	0.006	0.005	16913	-0.095	0.071	0.183	172940	0.081	0.004	6.0E-85	0.014	Yes
rs4148218	2:43953086	ABCG8	G/A	0.885	0.885	16913	-0.005	0.017	0.762	173032	0.044	0.005	3.0E-21	0.005	Yes
rs2710642	2:63003061	EHBP1	A/G	0.688	0.730	16913	0.026	0.012	0.028	172994	0.024	0.004	3.0E-10	0.895	Yes
rs2030746	2:121025958	None	T/C	0.469	0.496	16913	0.002	0.011	0.873	173024	0.021	0.004	2.0E-08	0.089	Yes
rs16831243	2:135478814	YSK4	T/C	0.407	0.385	16913	0.003	0.011	0.809	162945	0.038	0.006	8.0E-12	0.005	Yes
rs2287623	2:169538401	ABCB11	G/A	0.254	0.260	16913	0.033	0.012	0.008	170077	0.022	0.004	7.0E-09	0.392	Yes
rs1250229	2:216012629	*FN1	C/T	0.932	0.923	16913	0.025	0.023	0.278	173032	0.024	0.004	8.0E-09	0.982	Yes
rs11563251	2:234344123	UGT1A1/UGT1A10/UGT1A3/UGT1A4/UGT1A5/UGT1A6/UGT1A7/UGT1A8/UGT1A9	T/C	0.085	0.118	16913	0.012	0.020	0.557	172855	0.035	0.006	2.0E-08	0.280	Yes
rs9875338	3:12271469	*GSTM1L/PPARG	G/A	0.918	0.931	16913	0.037	0.020	0.060	172895	0.027	0.004	3.0E-13	0.613	Yes
rs17345563	3:133691893	DNAJC13	A/G	0.898	0.882	16913	0.014	0.018	0.430	173048	0.036	0.006	3.0E-10	0.262	Yes
rs7703051	5:74661243	*COL4A3BP/HMGCR	A/C	0.528	0.510	16913	0.078	0.011	7.2E-13	173015	0.073	0.004	5.0E-85	0.636	Yes
rs6882076	5:156322875	*TIMD4	C/T	0.733	0.727	16913	0.055	0.012	8.0E-06	173006	0.046	0.004	5.0E-33	0.469	Yes
rs2294261	6:16217142	*MYLIP	A/C	0.097	0.083	16913	0.009	0.018	0.640	171831	0.033	0.004	5.0E-19	0.191	Yes
rs1800562	6:26201120	HFE	G/A	0.999	1.000	10871	-0.012	0.177	0.947	171209	0.062	0.008	2.0E-14	0.679	Yes
rs2247056	6:31373469	HLA-B/HLA-B*0707	C/T	0.853	0.834	16913	-0.010	0.015	0.491	169288	0.025	0.004	6.0E-09	0.026	Yes
rs868943	6:116444196	FRK	G/A	0.953	0.955	16913	-0.005	0.025	0.837	170111	0.026	0.004	1.0E-12	0.218	Yes

						CKB Univariate Model				GLGC (As Used for Score)					
rsID	Chr:Pos	Genes	Effect/ other allele	EAF (CKB)	EAF (1KG)	N	Estimate (SD)	SE	P-value	N	Estimate (SD)	SE	P-value	Het P-value	Included in CKB LDL-C GS?
rs1564348	6:160498850	SLC22A1	C/T	0.004	0.004	16335	0.100	0.086	0.242	172989	0.048	0.005	3.0E-22	0.543	Yes
rs12670798	7:21573877	DNAH11	C/T	0.491	0.502	16913	0.015	0.011	0.175	173013	0.034	0.004	7.0E-16	0.092	Yes
rs4722551	7:25958351	None	C/T	0.029	0.019	16913	0.036	0.030	0.239	172946	0.039	0.005	7.0E-16	0.916	Yes
rs217386	7:44567220	*DDX21/DDX56/DQ574505/NPC1L1/OGDH/TMED4	G/A	0.968	0.976	16913	0.017	0.030	0.559	173021	0.036	0.004	8.0E-22	0.529	Yes
rs4240624	8:9221641	AK055863	A/G	0.988	0.988	16913	0.146	0.051	0.004	171657	0.067	0.006	7.0E-27	0.122	Yes
rs10102164	8:55584167	*SOX17	A/G	0.214	0.207	16913	0.036	0.013	0.007	173037	0.032	0.005	3.0E-12	0.775	Yes
rs2326077	8:59548473	*CYP7A1/UBXN2B	C/T	0.205	0.244	16913	0.041	0.014	0.002	172996	0.034	0.004	2.0E-19	0.619	Yes
rs2737252	8:116733072	TRPS1	G/A	0.699	0.703	16913	0.035	0.012	0.003	172950	0.031	0.004	1.0E-14	0.757	Yes
rs2954022	8:126551803	*TRIB1	C/A	0.430	0.445	16913	0.050	0.011	4.7E-06	172992	0.055	0.004	4.0E-51	0.718	Yes
rs7832643	8:145094645	PLEC1	T/G	0.153	0.148	16913	-0.003	0.016	0.845	164854	0.034	0.004	7.0E-19	0.021	Yes
rs3780181	9:2630759	VLDLR	A/G	0.910	0.887	16913	0.049	0.020	0.017	171976	0.045	0.007	1.0E-09	0.850	Yes
rs1883025	9:106704122	ABCA1	C/T	0.783	0.757	16913	0.017	0.013	0.207	172330	0.030	0.004	1.0E-11	0.363	Yes
rs579459	9:135143989	*ABO/SURF6	C/T	0.216	0.190	16913	0.084	0.013	1.7E-10	172706	0.067	0.005	3.0E-49	0.215	Yes
rs2255141	10:113923876	GPAM/RP11-426E5.2	A/G	0.312	0.242	16913	0.051	0.012	9.3E-06	173003	0.030	0.004	7.0E-14	0.080	Yes
rs174532	11:61305450	C11orf9	A/G	0.001	0.001	12601	-0.017	0.123	0.890	172981	0.035	0.004	5.0E-17	0.671	Yes
rs1535	11:61354548	FADS2	A/G	0.563	0.434	16913	0.045	0.011	9.2E-05	168360	0.053	0.004	3.0E-43	0.501	Yes
rs11220462	11:125749162	ST3GAL4	A/G	0.347	0.377	16913	0.029	0.011	0.011	145030	0.059	0.006	3.0E-23	0.018	Yes
rs653178	12:110492139	ATXN2	T/C	0.997	0.997	14981	0.103	0.080	0.195	165312	0.023	0.004	2.0E-09	0.312	Yes
rs6489818	12:110794963	MAPKAPK5	A/G	0.882	0.847	16913	-0.009	0.016	0.583	173044	0.028	0.005	6.0E-09	0.031	Yes
rs1169288	12:119901033	HNF1A	C/A	0.396	0.389	16913	0.017	0.011	0.142	163086	0.038	0.004	9.0E-21	0.088	Yes
rs4942486	13:31851388	BRCA2	T/C	0.462	0.467	16913	0.037	0.011	7.9E-04	171930	0.024	0.004	3.0E-11	0.283	Yes
rs8017377	14:23953727	KIAA1305	A/G	0.055	0.050	16913	0.055	0.024	0.024	172866	0.030	0.004	3.0E-15	0.317	Yes
rs9989419	16:55542640	*CETP/HERPUD1/NLRC5/SLC12A3	A/G	0.236	0.244	16913	-0.015	0.013	0.253	163625	0.028	0.004	8.0E-13	0.002	Yes
rs2000999	16:70665594	DLP/HPR	A/G	0.265	0.310	16913	0.041	0.013	9.9E-04	171510	0.065	0.005	1.0E-45	0.078	Yes
rs314253	17:7032374	*ACADVL/ASGR1/CS266489/DLG4/DVL2/PHF23	T/C	0.516	0.548	16913	0.029	0.011	0.008	169706	0.024	0.004	2.0E-10	0.672	Yes
rs4791641	17:8101874	PFAS	C/T	0.749	0.773	16913	-0.011	0.013	0.377	171304	0.020	0.004	4.0E-08	0.017	Yes
rs7225700	17:42746803	*AX748120/C17orf57/ITGB3	C/T	0.669	0.651	16913	0.014	0.012	0.247	171505	0.030	0.004	8.0E-15	0.187	Yes
rs6511720	19:11063306	LDLR	G/T	0.993	0.988	16913	0.192	0.064	0.002	170608	0.221	0.006	3.0E-289	0.651	Yes

						CKB Univariate Model				GLGC (As Used for Score)					
rsID	Chr:Pos	Genes	Effect/ other allele	EAF (CKB)	EAF (1KG)	N	Estimate (SD)	SE	P-value	N	Estimate (SD)	SE	P-value	Het P-value	Included in CKB LDL-C GS?
rs688	19:11088602	LDLR	T/C	0.167	0.184	16913	0.077	0.014	1.1E-07	166792	0.054	0.004	9.0E-48	0.130	Yes
rs7254892	19:50081436	PVRL2	G/A	0.934	0.916	16913	0.703	0.021	5.7E-236	139198	0.485	0.012	3.8E-357	2.0E-19*	Yes
rs492602	19:53898229	FUT2	G/A	0.007	0.004	16913	0.047	0.059	0.427	170009	0.029	0.004	3.0E-14	0.763	Yes
rs364585	20:12910718	*SPTLC3	G/A	0.579	0.567	16913	-0.009	0.011	0.390	171526	0.025	0.004	4.0E-11	0.003	Yes
rs2328223	20:17793921	None	C/A	0.208	0.225	16913	0.020	0.013	0.140	170762	0.030	0.005	2.0E-09	0.484	Yes
rs7264396	20:33618155	FER1L4	C/T	0.776	0.782	16913	0.008	0.013	0.522	171527	0.025	0.005	3.0E-08	0.241	Yes
rs6016381	20:38613850	None	T/C	0.424	0.407	16913	0.019	0.011	0.090	171556	0.036	0.004	6.0E-22	0.147	Yes
rs6065311	20:39157752	TOP1	C/T	0.820	0.806	16913	0.018	0.014	0.209	171333	0.042	0.004	3.0E-30	0.102	Yes
rs1800961	20:42475778	HNF4A	C/T	0.985	0.984	16913	0.027	0.044	0.541	142698	0.069	0.011	1.0E-10	0.362	Yes
rs5763662	22:28708703	MTMR3	T/C	0.096	0.115	16913	0.025	0.019	0.189	162777	0.077	0.012	2.0E-10	0.020	Yes
rs1010167	1:110000250	GSTM2/GSTM4	G/C	0.654	0.639	16913	-0.019	0.012	0.109	165771	0.025	0.004	3.0E-10	3.7E-04*	No
rs515135	2:21139562	*APOB	C/T	0.897	0.917	16913	-0.009	0.018	0.604	173029	0.139	0.005	1.0E-188	8.9E-16*	No
rs7640978	3:32508014	CMTM6	C/T	0.942	0.930	16913	-0.047	0.023	0.044	172228	0.039	0.007	1.0E-08	3.9E-04*	No
rs4530754	5:122883315	CSNK1G3	A/G	0.352	0.343	16913	-0.015	0.011	0.187	173003	0.028	0.004	4.0E-14	3.5E-04*	No
rs2297374	6:160495975	SLC22A1	C/T	0.658	0.664	16913	-0.013	0.012	0.263	172978	0.033	0.004	6.0E-18	1.8E-04*	No
rs2073547	7:44548856	*DDX21/DDX56/DQ574505/NPC1L1/TMED4	G/A	0.369	0.375	16913	-0.003	0.011	0.780	169889	0.049	0.005	5.0E-23	2.6E-05*	No
rs10832962	11:18612847	*AK097654/AL137366/SPTY2D1/UEVLD	T/C	0.489	0.491	16913	-0.010	0.011	0.363	172920	0.032	0.004	2.0E-15	3.0E-04*	No
rs10790162	11:116144314	BUD13	A/G	0.212	0.235	16913	-0.034	0.013	0.011	173007	0.076	0.007	3.0E-26	3.7E-13*	No
rs10401969	19:19268718	SF4	T/C	0.906	0.893	16913	-0.034	0.019	0.075	171477	0.118	0.007	2.0E-60	7.4E-14*	No
rs6859	19:50073874	PVRL2	A/G	0.315	0.309	16913	-0.040	0.012	6.5E-04	166756	0.084	0.004	1.0E-101	1.1E-22*	No

CKB estimates are for rank inverse normal transformed LDL-C, after adjustment for age, age^2, fasting time, fasting time^2, ascertainment group, sex, and 1-9 regional principal components, stratified by region.
Exclusion criteria: Bonferroni corrected heterogeneity p-value < 0.05/74 and either non-significant CKB association (p-value > 0.05/74) OR non-concordant direction of effect for the CKB lipid effect estimate
* in genes column indicates genes were found within 50KB, used where no genes overlap SNP location
* in Het P-value column indicates significant heterogeneity after Bonferroni correction

Supplementary Table 4: ICD-10 codes of vascular and non-vascular endpoints in CKB

Vascular outcomes

- Major coronary events (MCE): Fatal IHD (ICD-10: I20-I25) or non-fatal myocardial infarction (ICD-10: I21) or coronary revascularisation procedures.
- Fatal or non-fatal myocardial infarction (ICD-10: I21).
- Fatal or non-fatal ischaemic stroke (ICD-10: I63).
- Fatal or non-fatal intracerebral haemorrhage (ICD-10: 161).
- Fatal or non-fatal stroke (ICD-10: I60, I61, 163, I64).
- Occlusive CVD: Fatal IHD (ICD-10: I20-I25) or non-fatal myocardial infarction (ICD-10: I21) or coronary revascularisation procedures or fatal or non-fatal ischaemic stroke (ICD-10: I63).
- Fatal cardiovascular disease (ICD-10: I00–99).
- Major vascular events (MVE): Fatal or non-fatal myocardial infarction (ICD-10: I21) or coronary revascularisation procedures or fatal or non-fatal stroke (ICD-10: I60, I61, 163, I64) or fatal cardiovascular disease (ICD-10: I00–I99).
- **Common vascular controls**: Exclude prevalent CHD, prevalent stroke or transient ischaemic attack or incident MVE.

Main non-vascular outcomes

- Diabetes (ICD-10: E10 – E14), including prevalent and screen-detected diabetes.
- Chronic kidney disease (CKD) (ICD-10: E10.2+, E11.2+, E12.2+, E13.2+, E14.2+, I12.0, I12.9, I13.0, I13.1, I13.2, I13.9, M10.3, M32.1+, N02, N03, N04, N05, N08.3*, N11, N12, N13, N15, N18, N19, N25, N26, N27.1, N27.9, N28.9, O10.2, O10.3, R94.4, T86.1, Z94.0). Exclude prevalent kidney disease from controls.
- Chronic liver disease including cirrhosis and hepatitis (ICD-10: CD-10 B18, B19, B25.1+, B58.1+, B94.2, K72, K73, K74, K75, K76, K77*, Z22.5) Exclude prevalent cirrhosis or hepatitis from controls.
- Malignant neoplasms (ICD-10: C00-C97). Exclude prevalent cancer from controls.
- Eye diseases (ICD-10: H00-H59).
- COPD (ICD-10: J41-J44)
- Non-vascular mortality (all ICD-10 codes excluding I00-I99).

Respiratory outcomes

- Chronic obstructive pulmonary disease (COPD)
    - Incident COPD: ICD-10: J41 –J44.
    - Prevalent COPD: COPD at baseline according to spirometry measures and Global Lung Initiative definition of FEV1/FVC less than lower limit of normal.
- COPD Exacerbation: Incident events in individuals with COPD at baseline.
- Upper Respiratory Tract Infection (URTI): J00-J06, first event occuring 2009 or later.

Supplementary Table 5: ICD-10 codes of endpoints included in the phenome scan

Endpoint description (full)	Endpoint description (short)	ICD-10 Codes
Certain infections and parasitic diseases	Infectious and parasitic diseases	A00-B99
Malignant neoplasms (excluding bronchus and lung)	Malignant neoplasms (excluding bronchus and lung),	C00-C33, C37-C99
Malignant neoplasms of bronchus and lung	Malignant neoplasms of bronchus and lung,	C34
In situ, benign, uncertain or unknown behaviour neoplasms	In situ, benign, uncertain or unknown behaviour neoplasms	D00-D48
Diseases of the blood and blood-forming organs and certain disorders involving the immune mechanism	Diseases of the blood	D50-D89
Disorders of thyroid gland, Other disorders of glucose regulation and pancreatic internal secretion, Disorders of other endocrine glands	Other endocrine disorders	E00-E07, E15-E35
Diabetes mellitus	Diabetes	E10-E14
Malnutrition, Other nutritional deficiencies, Obesity and other hyperalimentation, Metabolic disorders	Malnutrition or other nutritional deficiencies	E40-E77, E79-E90
Disorders of lipoprotein metabolism and other lipidaemias	Disorders of lipoprotein metabolism	E78
Mental and behavioural disorders	Mental and behavioural disorders,	F00-F99
Diseases of the nervous system (excluding TIA)	Diseases of the nervous system	G00-G44, G46-G99
Transient cerebral ischaemic attacks and related syndromes	Transient cerebral ischaemic attacks	G45
Diseases of the eye and adnexa	Diseases of the eye and adnexa	H00-H59
Diseases of the ear and mastoid process	Diseases of the ear and mastoid process	H60-H95
Diseases of the circulatory system (excluding hypertension, IHD, cerebrovascular disease)	Diseases of the circulatory system	I00-I09, I11-I15, I26-I52, I70-I99
Essential (primary) hypertension	Essential hypertension	I10
Ischaemic heart diseases	Ischaemic heart disease	I120-I25
Cerebrovascular diseases	Cerebrovascular disease	I60-I69
Acute upper respiratory infections	Acute Upper Respiratory Infections	J00-J06
Influenza and pneumonia	Influenza and pneumonia	J09-J18
Other acute lower respiratory infections	Other acute lower respiratory infections	J20-J22
Other diseases of upper respiratory tract	Other diseases of upper respiratory tract	J30-J39

Endpoint description (full)	Endpoint description (short)	ICD-10 Codes
Chronic lower respiratory diseases (excluding asthma)	Chronic lower respiratory disease	J40-J44, J47
Asthma, status asthmaticus	Asthma	J45-J46
Other diseases of the respiratory system	Other diseases of the respiratory system,	J60-J99
Diseases of oral cavity, salivary glands and jaws (excluding gingivitis and periodontal diseases)	Diseases of oral cavity	K00-K04, K06-K14
Gingivitis and periodontal diseases	Gingivitis and periodontal diseases	K05
Diseases of oesophagus, stomach and duodenum	Diseases of oesophagus, stomach and duodenum	K20-K31
Diseases of appendix, Hernia, Other diseases of intestines, Diseases of peritoneum, Other diseases of the digestive system	Other digestive system diseases	K35-K46, K55-K67, K90-K93
Crohn disease [regional enteritis], Ulcerative colitis, Other noninfective gastroenteritis and colitis	Noninfective enteritis and colitis	K50-K52
Diseases of liver	Diseases of the liver	K70-K77
Disorders of gallbladder, biliary tract and pancreas	Disorders of biliary tract and pancreas	K80-K87
Diseases of the skin and subcutaneous tissue	Diseases of the skin	L00-L99
Arthropathies	Arthropathies	M00-M25
Diseases of the musculoskeletal system and connective tissue (excluding arthropothies)	Other musculoskeletal disorders	M30-M99
Glomerular diseases, Renal tubulo-interstitial diseases, Renal failure, Urolithiasis, Other disorders of kidney and ureter	Renal disorders	N00-N29
Other diseases of urinary system	Other diseases of urinary system	N30-N39
Diseases of male genital organs	Diseases of the male genital organs	N40-N51
Disorders of breast	Disorders of breast	N60-N64
Inflammatory diseases of female pelvic organs	Inflammatory diseases of female pelvic organs	N70-N77
Non-inflammatory disorders of female genital tract, Other disorders of the genitourinary system	Other female genitourinary diseases	N80-N99

Supplementary Table 6: Numbers and ascertainments of cases and controls included in analyses of disease associations with the *PCSK9* genetic score

		**Ascertainment Group**								
		**Population Representative**	**IS case**	**MI case**	**Fatal IHD case**	**ICH case**	**SAH case**	**COPD**	**Other**	**Total**
**Group**	**Endpoint**	**Cases/ Controls**	**Cases/ Controls**	**Cases/ Controls**	**Cases/ Controls**	**Cases/ Controls**	**Cases/ Controls**	**Cases/ Controls**	**Cases/ Controls**	**Cases/ Controls**
Figure 2: Cardiovascular diseases	Major Coronary Events	2390/59421	X/X	1657/X	696/X	X/X	X/X	X/X	277/X	5020/59421
	Ischaemic Stroke	6938/59421	4064/X	X/X	X/X	X/X	X/X	X/X	465/X	11467/59421
	Intracerebral haemorrhage	1102/59421	X/X	X/X	X/X	4680/X	X/X	X/X	124/X	5906/59421
	Major Occlusive Vascular Events	8631/59421	4064/X	1657/X	696/X	X/X	X/X	X/X	704/X	15752/59421
	Fatal Occlusive Vascular Events	1049/59421	118/X	961/X	696/X	X/X	X/X	X/X	261/X	3085/59421
	Fatal Cardiovascular Disease	2161/59421	118/X	961/X	696/X	2340/X	44/X	X/X	323/X	6643/59421
	Major Vascular Events	9643/59421	4064/X	1657/X	696/X	4680/X	372/X	X/X	867/X	21979/59421
Figure 2: Other diseases	Diabetes	6863/64051	X/X	X/X	X/X	X/X	X/X	X/X	605/X	7468/64051
	Chronic Kidney Disease	963/68918	X/X	X/X	X/X	X/X	X/X	X/X	117/X	1080/68918
	Chronic Liver Disease	607/69492	X/X	X/X	X/X	X/X	X/X	X/X	72/X	679/69492
	Malignant Neoplasms	4099/66593	X/X	X/X	X/X	X/X	X/X	X/X	462/X	4561/66593
	Diseases of Eye and Adnexa	3114/67800	X/X	X/X	X/X	X/X	X/X	X/X	440/X	3554/67800
	Incident COPD	2559/66997	X/X	X/X	X/X	X/X	X/X	3727/X	550/X	6836/66997
	Non-Vascular Mortality	3758/67156	X/X	X/X	X/X	X/X	X/X	X/X	627/X	4385/67156
Figure 3: UKB repliication	Acute upper respiratory infections	980/69934	X/X	X/X	X/X	X/X	X/X	X/X	115/X	1095/69934
	Asthma diagnosed by doctor	389/70525	X/X	X/X	X/X	X/X	X/X	X/X	38/X	427/70525
	Mood (affective) disorders	132/70782	X/X	X/X	X/X	X/X	X/X	X/X	5/X	137/70782
	Dorsopathies not classified as deforming	2198/68716	X/X	X/X	X/X	X/X	X/X	X/X	244/X	2442/68716
	Cerebrovascular diseases	9681/61233	X/X	X/X	X/X	X/X	X/X	X/X	X/X	9681/61233
	Soft tissue disorders including bursitis	291/70623	X/X	X/X	X/X	X/X	X/X	X/X	39/X	330/70623
	Malignant neoplasm of breast	389/41954	X/X	X/X	X/X	X/X	X/X	X/X	25/X	414/41954

		Ascertainment Group								
		Population Representative	IS case	MI case	Fatal IHD case	ICH case	SAH case	COPD	Other	Total
Group	Endpoint	Cases/ Controls	Cases/ Controls	Cases/ Controls	Cases/ Controls	Cases/ Controls	Cases/ Controls	Cases/ Controls	Cases/ Controls	Cases/ Controls
Figure 4: Respiratory Disease	Prevalent COPD	4539/52355	X/X	X/X	X/X	X/X	X/X	X/X	566/X	5105/52355
	Incident COPD	2559/66997	X/X	X/X	X/X	X/X	X/X	3727/X	550/X	6836/66997
	Incident COPD (Fatal)	511/66997	X/X	X/X	X/X	X/X	X/X	971/X	150/X	1632/66997
	Incident COPD (Non-Fatal)	2048/66997	X/X	X/X	X/X	X/X	X/X	2756/X	400/X	5204/66997
	COPD Exacerbations	754/3785	X/X	X/X	X/X	X/X	X/X	1304/X	154/412	2212/4197
	COPD Exacerbations (Fatal)	208/3785	X/X	X/X	X/X	X/X	X/X	476/X	46/412	730/4197
	COPD Exacerbations (Non-Fatal)	546/3785	X/X	X/X	X/X	X/X	X/X	828/X	108/412	1482/4197
	Acute upper respiratory infections	980/69934	X/X	X/X	X/X	X/X	X/X	X/X	115/X	1095/69934
Figure S7: Phenome-wide Scan	Infectious and parasitic diseases	2232/68682	X/X	X/X	X/X	X/X	X/X	X/X	343/X	2575/68682
	Malignant neoplasms (not bronchus/lung)	3492/67422	X/X	X/X	X/X	X/X	X/X	X/X	365/X	3857/67422
	Malignant neoplasms (bronchus/lung)	884/70030	X/X	X/X	X/X	X/X	X/X	X/X	125/X	1009/70030
	In situ, benign, uncertain or unknown behaviour neoplasms	2034/68880	X/X	X/X	X/X	X/X	X/X	X/X	144/X	2178/68880
	Diseases of the blood	595/70319	X/X	X/X	X/X	X/X	X/X	X/X	68/X	663/70319
	Other endocrine disorders	645/70269	X/X	X/X	X/X	X/X	X/X	X/X	42/X	687/70269
	Diabetes mellitus	4971/65943	X/X	X/X	X/X	X/X	X/X	X/X	424/X	5395/65943
	Malnutrition/other nutritional deficiencies	446/70468	X/X	X/X	X/X	X/X	X/X	X/X	67/X	513/70468
	Disorders of lipoprotein metabolism	535/70379	X/X	X/X	X/X	X/X	X/X	X/X	45/X	580/70379
	Mental and behavioural disorders,	730/70184	X/X	X/X	X/X	X/X	X/X	X/X	70/X	800/70184
	Diseases of the nervous system	1347/69567	X/X	X/X	X/X	X/X	X/X	X/X	134/X	1481/69567
	Transient cerebral ischaemic attacks	2992/67922	X/X	X/X	X/X	X/X	X/X	X/X	270/X	3262/67922
	Diseases of the eye and adnexa	3114/67800	X/X	X/X	X/X	X/X	X/X	X/X	440/X	3554/67800
	Diseases of the ear and mastoid process	697/70217	X/X	X/X	X/X	X/X	X/X	X/X	61/X	758/70217
	Diseases of the circulatory system	5082/65832	X/X	X/X	X/X	X/X	X/X	X/X	634/X	5716/65832
	Essential hypertension	5500/65414	X/X	X/X	X/X	X/X	X/X	X/X	673/X	6173/65414
	Ischaemic heart disease	7548/63366	X/X	X/X	X/X	X/X	X/X	X/X	779/X	8327/63366
	Cerebrovascular disease	9681/61233	X/X	X/X	X/X	X/X	X/X	X/X	869/X	10550/61233
	Acute upper respiratory infections	1126/69788	X/X	X/X	X/X	X/X	X/X	X/X	125/X	1251/69788

		Ascertainment Group								
		Population Representative	IS case	MI case	Fatal IHD case	ICH case	SAH case	COPD	Other	Total
Group	Endpoint	Cases/ Controls	Cases/ Controls	Cases/ Controls	Cases/ Controls	Cases/ Controls	Cases/ Controls	Cases/ Controls	Cases/ Controls	Cases/ Controls
	Influenza and pneumonia	3901/67013	X/X	X/X	X/X	X/X	X/X	X/X	534/X	4435/67013
	Other acute lower respiratory infections	1089/69825	X/X	X/X	X/X	X/X	X/X	X/X	150/X	1239/69825
	Other diseases of upper respiratory tract	635/70279	X/X	X/X	X/X	X/X	X/X	X/X	44/X	679/70279
	Chronic lower respiratory disease	3271/67643	X/X	X/X	X/X	X/X	X/X	X/X	617/X	3888/67643
	Asthma	353/70561	X/X	X/X	X/X	X/X	X/X	X/X	50/X	403/70561
	Other diseases of the respiratory system,	777/70137	X/X	X/X	X/X	X/X	X/X	X/X	113/X	890/70137
	Diseases of oral cavity	147/70767	X/X	X/X	X/X	X/X	X/X	X/X	8/X	155/70767
	Gingivitis and periodontal diseases	34/70880	X/X	X/X	X/X	X/X	X/X	X/X	6/X	40/70880
	Diseases of oesophagus, stomach and duodenum	2995/67919	X/X	X/X	X/X	X/X	X/X	X/X	397/X	3392/67919
	Other digestive system diseases	3032/67882	X/X	X/X	X/X	X/X	X/X	X/X	288/X	3320/67882
	Noninfective enteritis and colitis	220/70694	X/X	X/X	X/X	X/X	X/X	X/X	38/X	258/70694
	Diseases of the liver	829/70085	X/X	X/X	X/X	X/X	X/X	X/X	85/X	914/70085
	Disorders of biliary tract and pancreas	2470/68444	X/X	X/X	X/X	X/X	X/X	X/X	298/X	2768/68444
	Diseases of the skin	649/70265	X/X	X/X	X/X	X/X	X/X	X/X	86/X	735/70265
	Arthropathies	1461/69453	X/X	X/X	X/X	X/X	X/X	X/X	172/X	1633/69453
	Other musculoskeletal disorders	4689/66225	X/X	X/X	X/X	X/X	X/X	X/X	557/X	5246/66225
	Renal disorders	2621/68293	X/X	X/X	X/X	X/X	X/X	X/X	335/X	2956/68293
	Other diseases of urinary system	736/70178	X/X	X/X	X/X	X/X	X/X	X/X	92/X	828/70178
	Diseases of the male genital organs	653/27918	X/X	X/X	X/X	X/X	X/X	X/X	136/X	789/27918
	Disorders of breast (females only)	211/42132	X/X	X/X	X/X	X/X	X/X	X/X	10/X	221/42132
	Inflammatory diseases of female pelvic organs	573/41770	X/X	X/X	X/X	X/X	X/X	X/X	56/X	629/41770
	Other female genitourinary diseases	884/41459	X/X	X/X	X/X	X/X	X/X	X/X	55/X	939/41459

Supplementary Table 7: Disease definitions for replication of outcomes reported as associated with a functional *PCSK9* variant in UK Biobank

**Outcome description**	**Case definition**
Acute upper respiratory infections	ICD-10 J00-J06
Asthma diagnosed by doctor	self-report
Mood (affective) disorders	ICD-10 F30-F39
Dorsopathies not classified as deforming	ICD-10 M50-M54
Cerebrovascular diseases	ICD-10 I60-I69
Soft tissue disorders including bursitis	ICD-10 M70-M79
Malignant neoplasm of breast	ICD-10 C50, females only

Supplementary Table 8: Association of the *PCSK9* genetic score with carotid intima media thickness and presence of carotid plaque in CKB second resurvey participants

Phenotype	N	Effect (SD)*	SE	OR*	95% CI		P
					LL	UL	
Mean CIMT	20896	-0.24	0.06		–0.36	–0.12	0.00012
Plaque	8340 cases<br>12555 controls			0.61	0.45	0.83	0.0015

*Effect/OR expressed per 1 SD lower LDL-C

Supplementary Table 9: Association of the *PCSK9* genetic score with FEV1/FVC measured at baseline in CKB participants

SNP	Effect/ other allele	Effect allele freq.	N	effect (SD)*	SE	P-value
rs151193009	C/T	0.987	83715	-0.00483	0.00364	0.185
rs2495477	A/G	0.738	83715	0.01503	0.01088	0.167
rs11206517	G/T	0.060	83715	-0.00448	0.01096	0.683
Meta-analysis	-	-	-	-0.00298	0.00329	0.366

*Effect expressed per 1 SD lower LDL-C

Supplementary Table 10: Association of a loss-of-function *PCSK9* SNP with exacerbations of COPD in UK Biobank

SNP	Chr:bp	Effect/other allele	EAF	Per-allele log odds (SE) of COPD exacerbation	OR of COPD exacerbation per 1-SD lower LDL-C
rs11591147	1:55505647	G/T	0.983	0.005 (0.005)	0.55 (0.20, 1.50)

Supplementary Figures

Supplementary Figure 1. Study design flow-chart.

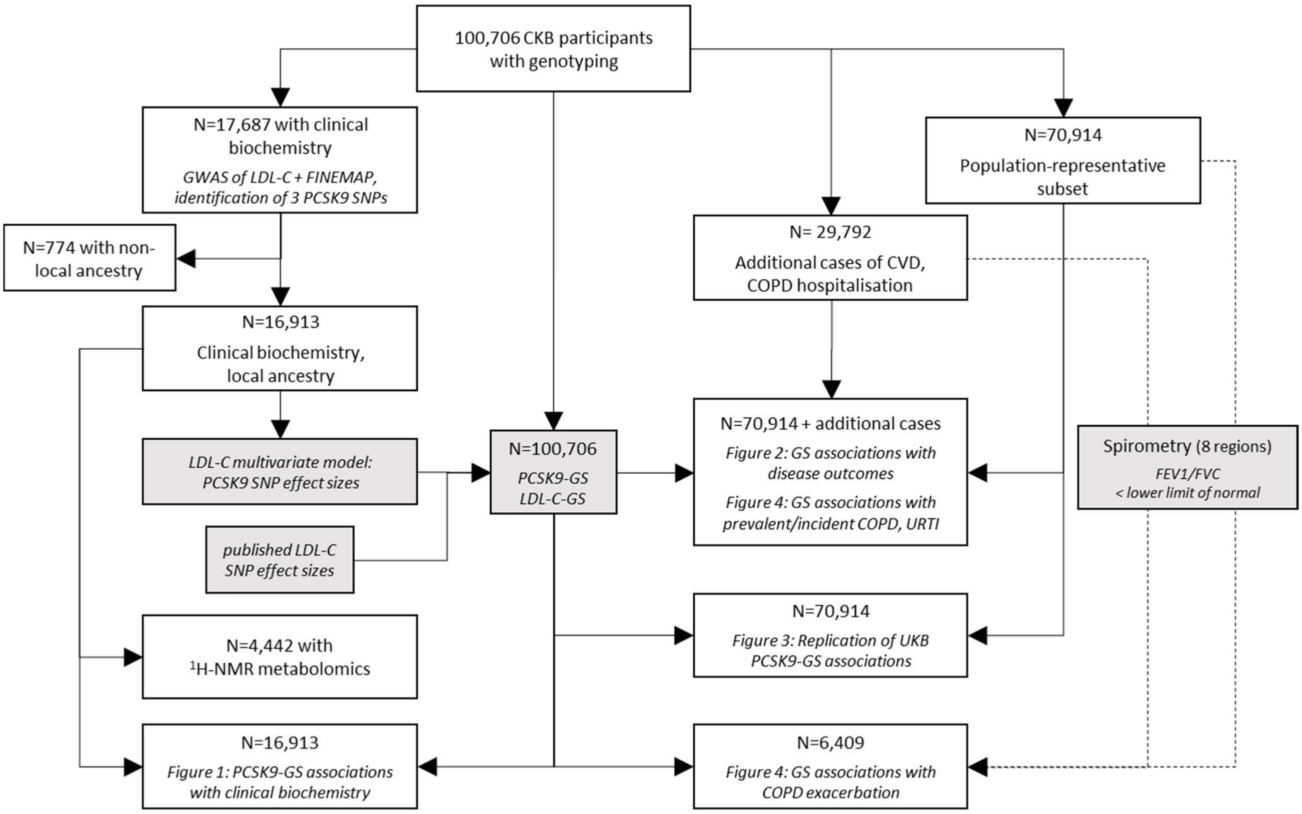

Supplementary Figure 2. Violin plots showing the distribution of measured FEV1/FVC in each CKB region

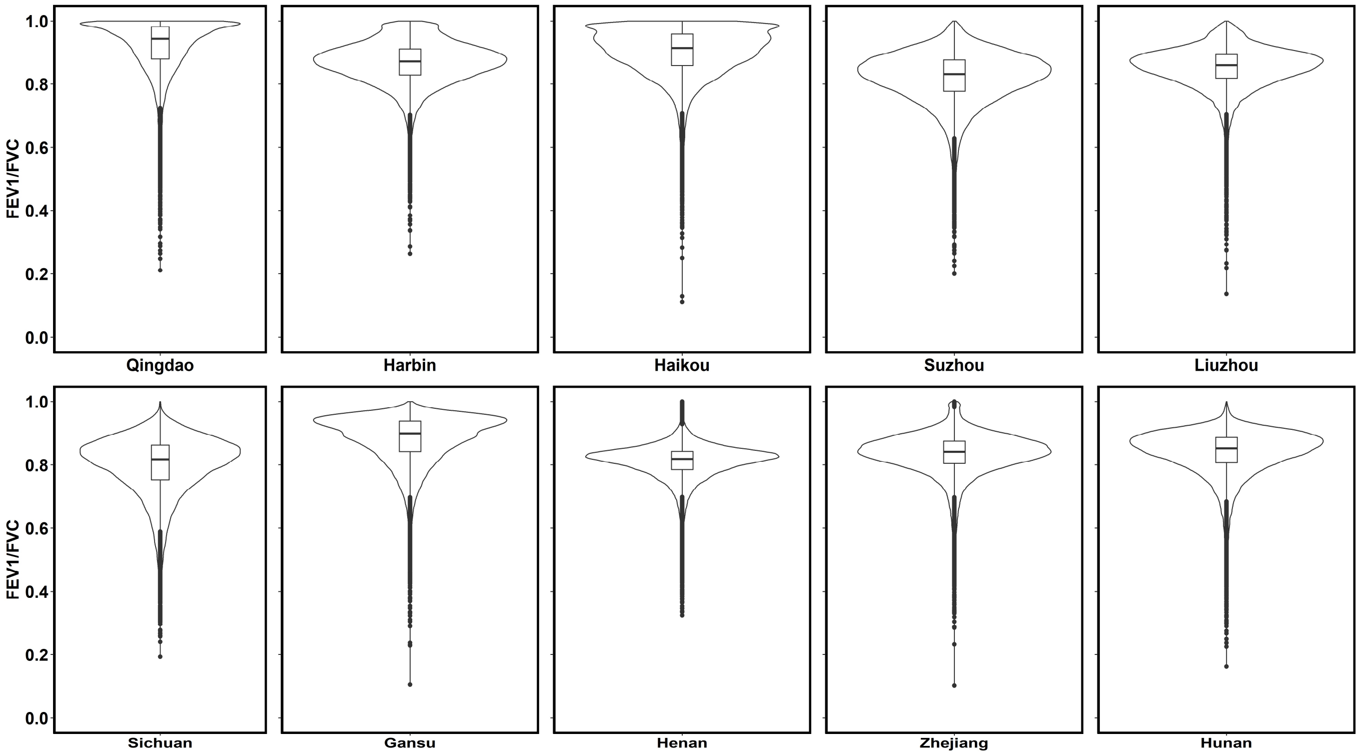

Supplementary Figure 3. Histogram of measured FEV1/FVC by prevalent COPD status.

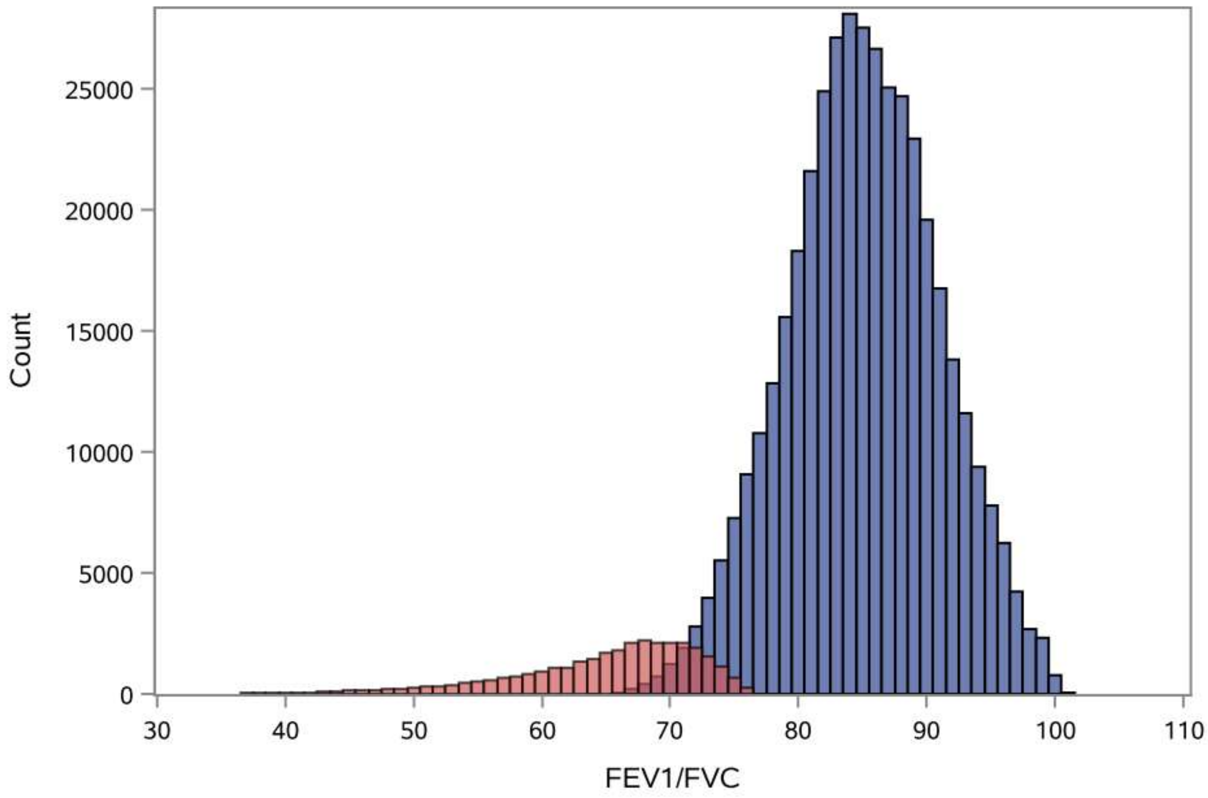

Red – CKB participants with COPD at baseline. Blue – CKB participants without COPD.
COPD defined according to FEV1/FVC below the lower limit of normal, as predicted from Global Lung Initiative reference equations[37], derived using ancestry, age, height, and sex.

Supplementary Figure 4: Frequency distribution of the *PCSK9* genetic score in the CKB population-representative subset.

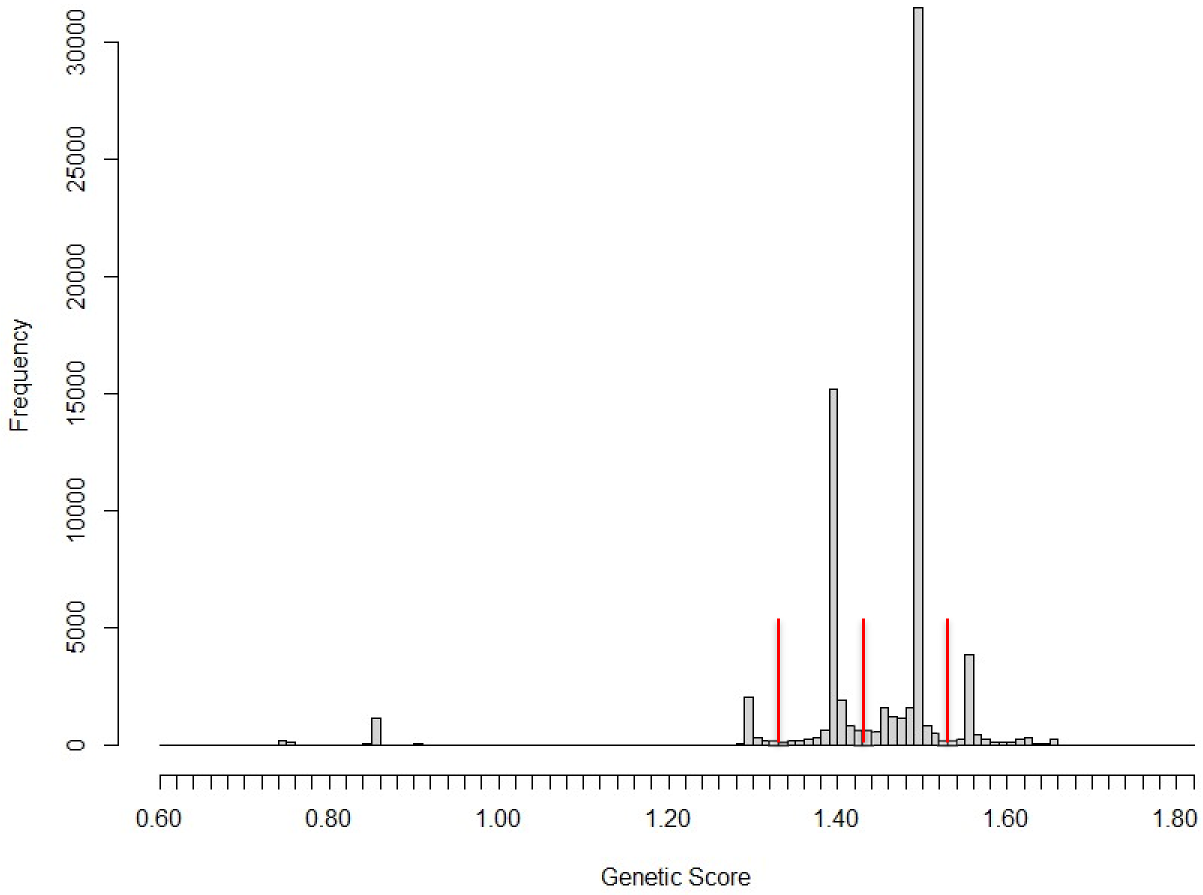

Supplementary Figure 5: Sub-group analyses of association of PCSK9 genetic score with LDL-cholesterol.

	N	Standardised difference (95% CI)	p	p het
**Region**				
Qingdao	578	-0.53 (-1.24, 0.18)	0.15	
Harbin	2250	-0.97 (-1.31, -0.62)	4.0×10^{-8}	
Haikou	405	-1.26 (-2.65, 0.12)	0.074	
Suzhou	640	-1.00 (-1.88, -0.12)	0.026	
Liuzhou	887	-1.42 (-2.32, -0.52)	0.0021	
Sichuan	1556	-1.28 (-1.77, -0.78)	3.9×10^{-7}	
Gansu	3062	-1.20 (-1.51, -0.88)	2.0×10^{-13}	
Henan	2958	-1.32 (-1.65, -0.99)	2.3×10^{-15}	
Zhejiang	1310	-1.09 (-1.60, -0.58)	2.6×10^{-5}	
Hunan	3267	-0.85 (-1.18, -0.53)	2.8×10^{-7}	0.49
**Urban/Rural**				
Rural	12153	-1.09 (-1.26, -0.93)	1.6×10^{-39}	
Urban	4760	-0.91 (-1.17, -0.64)	1.8×10^{-11}	0.24
**Sex**				
Male	8583	-0.99 (-1.18, -0.79)	2.8×10^{-23}	
Female	8330	-0.99 (-1.18, -0.81)	3.1×10^{-26}	0.96
**Age**				
<40	1210	-0.87 (-1.37, -0.37)	0.00063	
40-59	3183	-1.15 (-1.46, -0.84)	4.7×10^{-13}	
50-59	5540	-1.04 (-1.27, -0.81)	1.7×10^{-18}	
60-69	4972	-0.94 (-1.18, -0.70)	3.0×10^{-14}	
70+	2008	-0.79 (-1.21, -0.38)	0.00019	0.66
**Smoking status**				
No current regular smoking	11200	-1.01 (-1.17, -0.85)	7.7×10^{-35}	
Current regular smoking	5713	-0.98 (-1.22, -0.74)	2.8×10^{-15}	0.84
**Alcohol drinking**				
No regular drinking	14139	-1.00 (-1.15, -0.86)	4.9×10^{-42}	
Regular drinking	2774	-1.02 (-1.37, -0.67)	9.0×10^{-9}	0.93
**Prevalent COPD**				
No prevalent COPD	14460	-0.98 (-1.12, -0.83)	3.1×10^{-41}	
Prevalent COPD	1446	-1.23 (-1.77, -0.68)	9.8×10^{-6}	0.38
-				
**Overall**	16913	-1.00 (-1.13, -0.87)	2.1×10^{-48}	

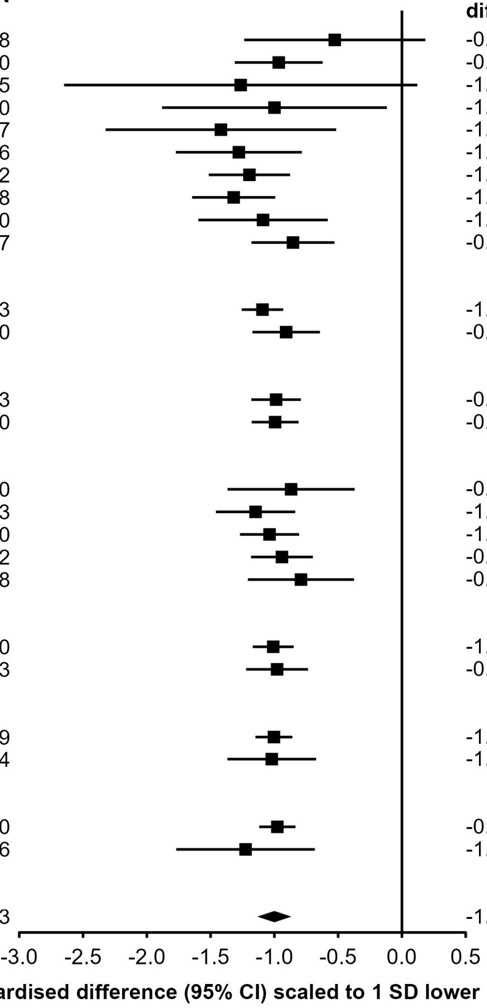

Analysis of prevalent COPD limited to 8 geographical regions in which spirometry passed quality control.

Supplementary Figure 6: Association of *PCSK9* genetic score with metabolic biomarkers quantified by ^{1}H-NMR spectroscopy.

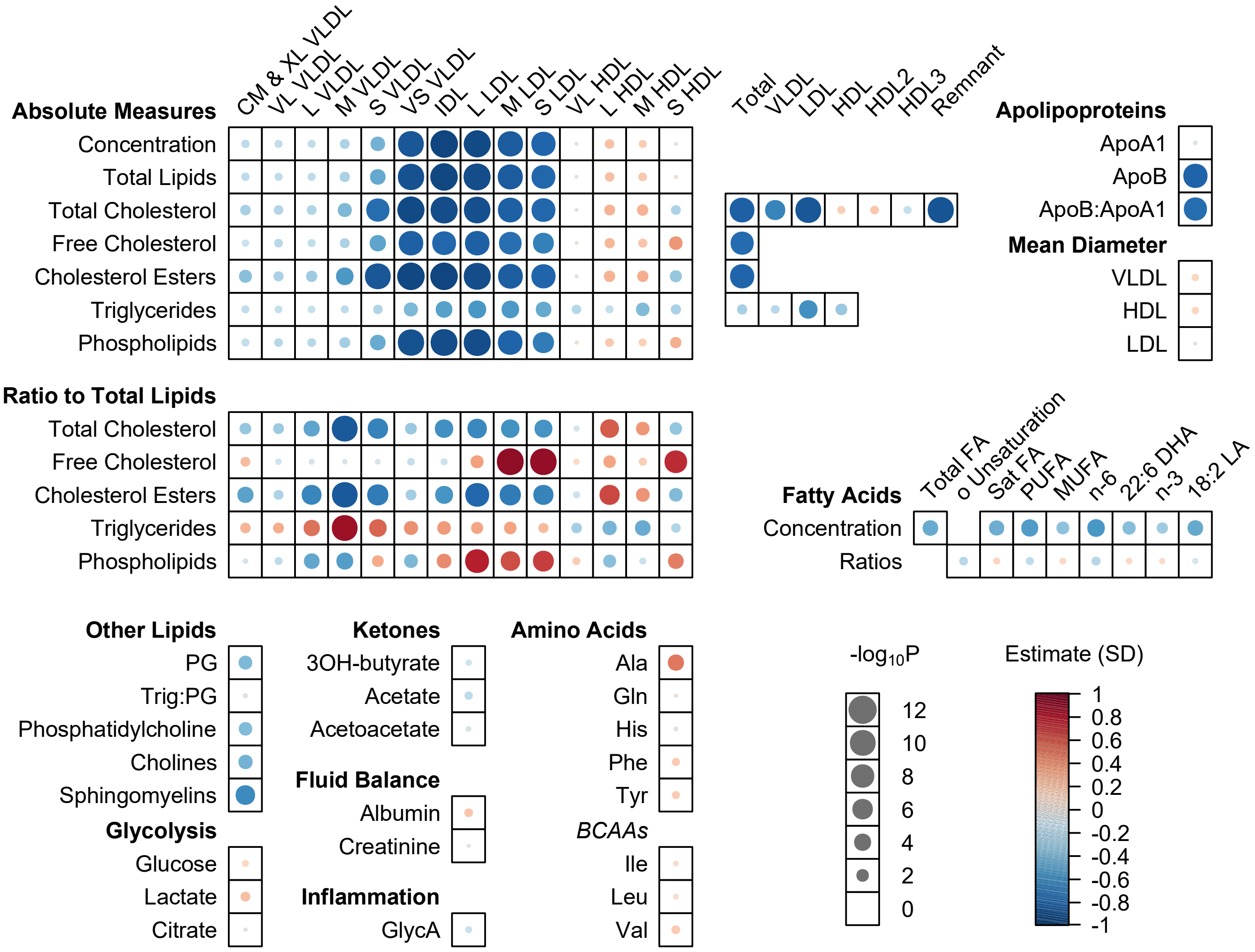

Heatmap representation of metabolome associations with the *PCSK9* genetic score was generated using the R package *bubblePlot*.

Supplementary Figure 7: Association of *PCSK9* genetic score with non-lipid blood measures

	N	Standardised difference (95% CI)	p
**Inflammation**			
C-reactive protein	16912	0.03 (-0.10, 0.17)	0.61
Fibrinogen	8765	-0.20 (-0.38, -0.01)	0.04
Glycoprotein acetyls	4441	-0.13 (-0.39, 0.12)	0.32
**Liver function**			
Alanine aminotransferase	16903	0.00 (-0.13, 0.13)	1
Aspartate aminotransferase	16907	-0.06 (-0.19, 0.07)	0.37
Gamma glutamyl transferase	15662	-0.04 (-0.18, 0.09)	0.53
Albumin	15838	0.11 (-0.03, 0.24)	0.13
**Renal function**			
Creatinine	16913	0.07 (-0.06, 0.21)	0.27
Uric acid	15656	0.10 (-0.04, 0.23)	0.17
Cystatin C	15330	0.08 (-0.06, 0.22)	0.25
**Other**			
25-OH vitamin D	8514	0.12 (-0.07, 0.31)	0.23

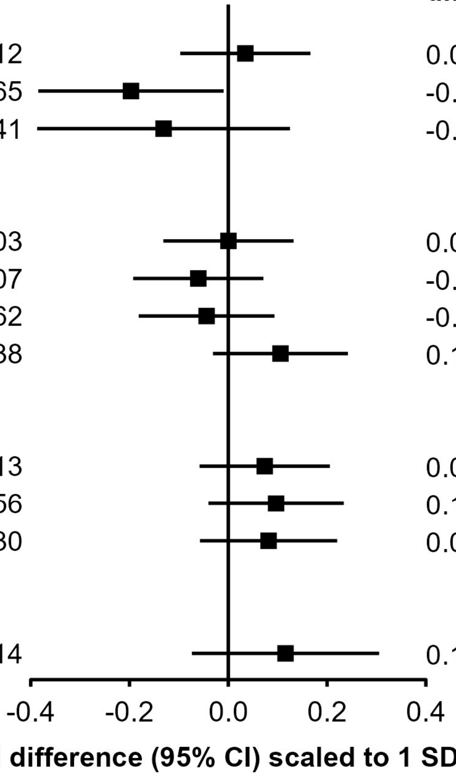

Glycoprotein acetyl measurements were derived from ^{1}H-NMR metabolomics, other measures were from clinical biochemistry assays.

Supplementary Figure 8: Phenome-wide scan of *PCSK9* genetic score associations in China Kadoorie Biobank

	Cases/Controls	OR (95% CI)	p
Infectious and parasitic diseases	2575/68682	1.28 (0.91, 1.81)	0.16
Malignant neoplasms (excluding bronchus and lung)	3857/67422	1.04 (0.78, 1.39)	0.77
Malignant neoplasms of bronchus and lung	1009/70030	1.09 (0.64, 1.87)	0.75
In situ, benign, uncertain or unknown behaviour neoplasms	2178/68880	0.91 (0.62, 1.35)	0.65
Diseases of the blood	663/70319	1.66 (0.88, 3.16)	0.12
Other endocrine disorders	687/70269	1.34 (0.71, 2.51)	0.37
Diabetes mellitus	5395/65943	0.94 (0.73, 1.21)	0.62
Malnutrition or other nutritional deficiencies	513/70468	1.06 (0.46, 2.42)	0.9
Disorders of lipoprotein metabolism	580/70379	0.69 (0.32, 1.51)	0.36
Mental and behavioural disorders	800/70184	1.04 (0.57, 1.93)	0.89
Diseases of the nervous system	1481/69567	1.37 (0.89, 2.10)	0.15
Transient cerebral ischaemic attacks	3262/67922	0.95 (0.70, 1.30)	0.76
Diseases of the eye and adnexa	3554/67800	0.98 (0.72, 1.33)	0.87
Diseases of the ear and mastoid process	758/70217	0.91 (0.47, 1.77)	0.78
Diseases of the circulatory system	5716/65832	1.11 (0.87, 1.41)	0.41
Essential hypertension	6173/65414	0.89 (0.70, 1.13)	0.34
Ischaemic heart disease	8327/63366	1.00 (0.81, 1.23)	0.99
Cerebrovascular disease	10550/61233	0.90 (0.74, 1.10)	0.3
Acute upper respiratory infections	1251/69788	1.98 (1.25, 3.13)	0.0034
Influenza and pneumonia	4435/67013	0.93 (0.70, 1.23)	0.61
Other acute lower respiratory infections	1239/69825	1.00 (0.59, 1.68)	0.99
Other diseases of upper respiratory tract	679/70279	1.17 (0.60, 2.28)	0.64
Chronic lower respiratory disease	3888/67643	1.08 (0.80, 1.48)	0.61
Asthma	403/70561	0.59 (0.22, 1.56)	0.29
Other diseases of the respiratory system	890/70137	1.06 (0.58, 1.92)	0.85
Diseases of oral cavity	155/70767	1.11 (0.24, 5.12)	0.89
Gingivitis and periodontal diseases	40/70880	0.21 (0.00, 9.74)	0.42
Diseases of oesophagus, stomach and duodenum	3392/67919	1.12 (0.82, 1.54)	0.47
Other digestive system diseases	3320/67882	1.12 (0.83, 1.53)	0.46
Noninfective enteritis and colitis	258/70694	3.25 (1.36, 7.74)	0.0079
Diseases of the liver	914/70085	1.10 (0.62, 1.96)	0.74
Disorders of biliary tract and pancreas	2768/68444	0.90 (0.63, 1.28)	0.57
Diseases of the skin	735/70265	1.02 (0.53, 1.94)	0.96
Arthropathies	1633/69453	1.16 (0.75, 1.81)	0.5
Other musculoskeletal disorders	5246/66225	1.13 (0.87, 1.45)	0.36
Renal disorders	2956/68293	0.73 (0.51, 1.04)	0.084
Other diseases of urinary system	828/70178	1.25 (0.70, 2.23)	0.45
Diseases of the male genital organs	789/27918	1.58 (0.86, 2.92)	0.14
Disorders of breast (female)	221/42132	3.34 (1.21, 9.22)	0.02
Inflammatory diseases of female pelvic organs	629/41770	1.03 (0.49, 2.16)	0.94
Other female genitourinary diseases	939/41459	1.08 (0.60, 1.95)	0.79

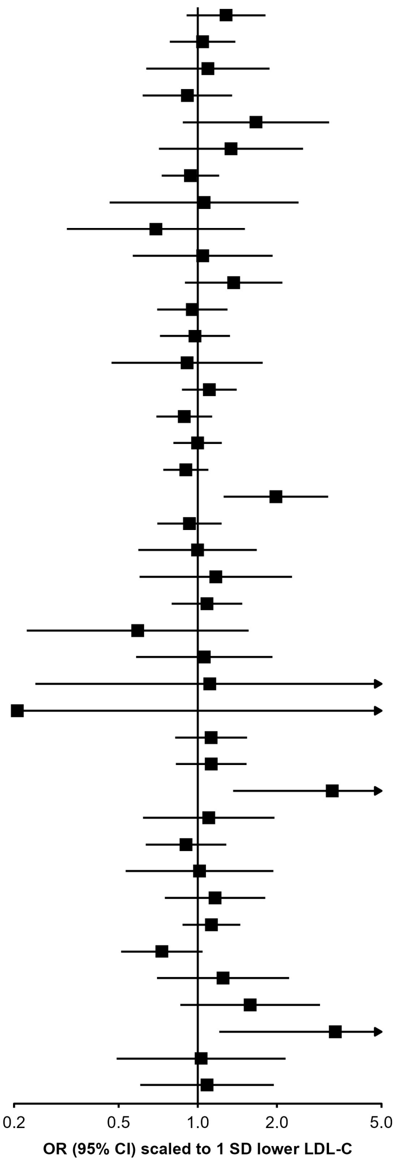

Supplementary Figure 9: Associations of *PCSK9*-GS with respiratory disease endpoints in CKB, with prevalent COPD defined as FEV1/FVC<0.7.

	Cases	Controls	OR (95% CI)	p
**Prevalent COPD**				
Overall	3856	60428	0.92 (0.67, 1.25)	0.58
**Incident COPD**				
Overall	6836	66997	1.38 (1.08, 1.76)	0.0089
Fatal	1632	66997	1.47 (0.93, 2.34)	0.098
Nonfatal	5204	66997	1.38 (1.05, 1.80)	0.019
**COPD Exacerbations**				
Overall	2238	2503	1.76 (1.00, 3.09)	0.049
Fatal	769	2503	3.53 (1.67, 7.47)	0.00094
Nonfatal	1469	2503	1.32 (0.69, 2.52)	0.4

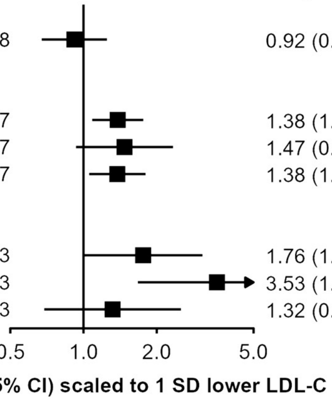

Supplementary Figure 10: Sub-group analyses of association of *PCSK9* genetic score with upper respiratory tract infections.

	Cases	Controls	OR (95% CI)	p	p het
**Urban/Rural**					
Rural	750	37779	2.33 (1.25, 4.35)	0.0075	
Urban	345	32155	1.18 (0.43, 3.20)	0.75	0.25
**Sex**					
Male	405	28226	2.09 (0.88, 4.93)	0.094	
Female	690	41708	1.82 (0.93, 3.55)	0.081	0.8
**Age**					
<40	114	10464	2.21 (0.49, 9.97)	0.3	
40-59	274	20646	1.11 (0.34, 3.64)	0.87	
50-59	346	21690	1.61 (0.60, 4.36)	0.35	
60-69	252	12683	3.09 (1.20, 7.95)	0.019	
70+	109	4451	1.61 (0.26, 9.75)	0.61	0.74
**Smoking status**					
No current regular smoking	832	51820	1.91 (1.05, 3.49)	0.034	
Current regular smoking	263	18114	1.82 (0.60, 5.50)	0.29	0.94
**Alcohol drinking**					
No regular drinking	923	59110	1.99 (1.13, 3.52)	0.018	
Regular drinking	172	10824	1.30 (0.32, 5.33)	0.71	0.58
**COPD at baseline**					
No	710	51645	2.10 (1.12, 3.93)	0.021	
Yes	109	4444	1.56 (0.29, 8.58)	0.61	0.75
–					
**Overall**	1095	69934	1.88 (1.11, 3.20)	0.019	

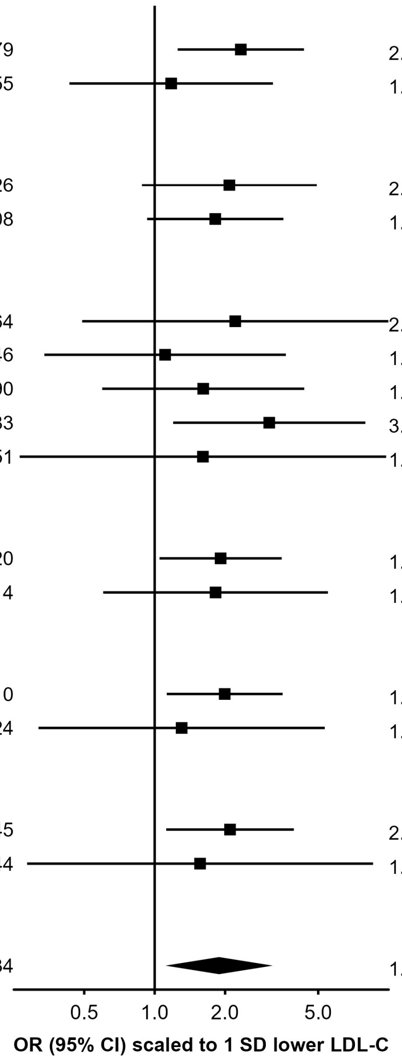

Analysis of prevalent COPD limited to 8 geographical regions in which spirometry passed quality control.
